# Paraneoplastic pemphigus associated with nonhuman papillomavirus–related tonsillar squamous cell carcinoma: A case report

**DOI:** 10.1097/MD.0000000000039368

**Published:** 2024-09-06

**Authors:** Shih-Chun Lu, Hung-Lun Chu, Hann-Ziong Yueh, Che-Hsuan Lin, Yang Chou

**Affiliations:** a Department of Otolaryngology, Taipei Medical University Hospital, Taipei, Taiwan; b Department of Otolaryngology, School of Medicine, College of Medicine, Taipei Medical University, Taipei, Taiwan; c Department of General Medicine, Taipei Medical University Hospital, Taipei, Taiwan

**Keywords:** human papillomavirus, oral ulcers, oropharyngeal cancer, paraneoplastic pemphigus, tonsillar cancer

## Abstract

**Background:**

Paraneoplastic pemphigus (PNP) is a rare, life-threatening autoimmune bullous disease. Among the ≈500 reported cases of PNP, only 1 case has been associated with tonsillar cancer, specifically, human papillomavirus (HPV)–positive squamous carcinoma. However, the occurrence of PNP in non-HPV–related tonsillar cancer is exceptionally rare and has not been reported to date.

**Methods:**

We present a 58-year-old male with a history of smoking, who experienced recurrent oral ulcers, right neck swelling, and hoarseness for 5 months. Diagnosis of right tonsillar squamous cell carcinoma (cT1N3bM0) was confirmed through computed tomography/magnetic resonance imaging and pathology, not associated with HPV. Histological and immunohistochemical findings indicated PNP.

**Results:**

The patient underwent primary tumor resection and ipsilateral neck dissection. Topical steroids and antifungal agents were administered to manage oral lesions and prevent secondary infections. Adjuvant concurrent chemoradiotherapy with cisplatin proceeded smoothly. Postconcurrent chemoradiotherapy follow-up at 3, 6, and 9 months, utilizing computed tomography/magnetic resonance imaging and nasopharyngoscopy, revealed no signs of recurrent cancer or PNP.

**Conclusion:**

Early indicators, such as oral mucosal ulcers and skin blisters, prompt consideration of underlying oral cancer in PNP. Comprehensive examination is crucial for diagnosing PNP and identifying concurrent internal neoplasms. Effective management includes occult malignancy treatment, postoperative steroid therapy, and infection prevention.

## 1. Introduction

Paraneoplastic pemphigus (PNP) is a rare autoimmune bullous disease with occult cancer, such as non-Hodgkin lymphoma (NHL) and chronic lymphocytic leukemia.^[1]^ PNP constitutes ≈3% to 5% of pemphigus cases and is characterized by a mortality rate of up to 90%.^[2]^ While autoimmune antibodies against plakin proteins are reported in PNP, a complete understanding of its pathogenesis remains unclear, posing challenges for early diagnosis. Tonsillar cancer is a well-known oral cancer, highly associated with risk factors, such as smoking, alcohol, betel nut, and human papillomavirus (HPV) infection.^[3]^ The association of PNP and tonsillar cancer is extremely rare. To the best of our knowledge, only 1 case report has documented the cooccurrence of tonsillar cancer and PNP, where the cancer was HPV p16 positive.^[4]^ To the best of our knowledge, PNP associated with non-HPV–related tonsil cancer has not been previously reported.

## 2. Case presentation

**Figure 1.**
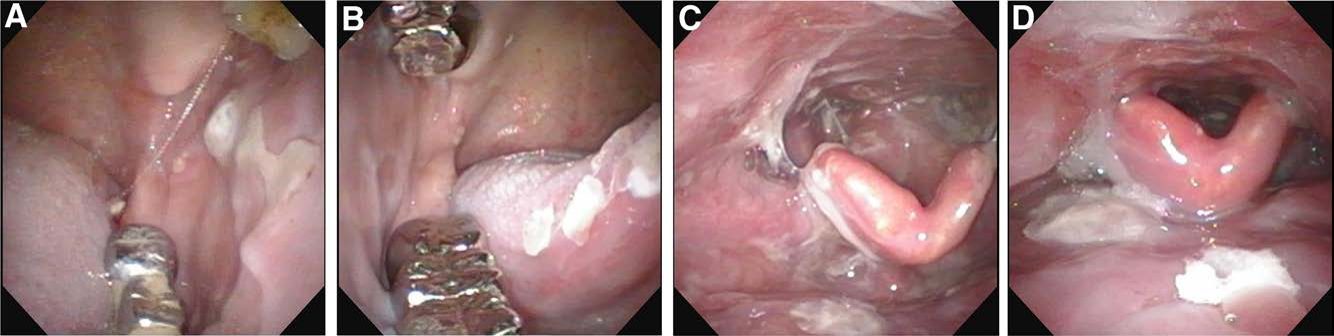
Multiple ulcers over (A) left buccal, (B) righty buccal, tongue, (C and D) tongue base, posterior pharyngeal wall, and epiglottis pyriform sinus.

**Figure 2.**
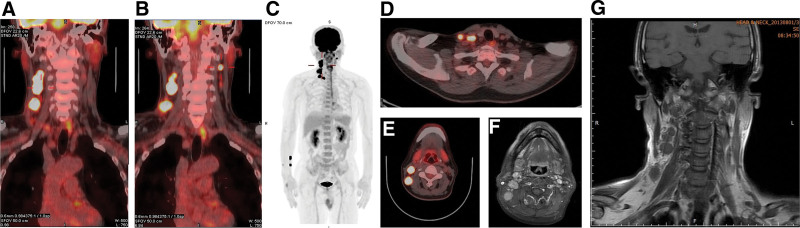
(A–E) The whole-body FDG-PET CT indicated right neck levels II to V enlarged lymphadenopathies and left neck levels II and III normal-sized lymphadenopathies. The FDG-avid pathologic lesions were not found in the gastrointestinal tract, liver, gallbladder, pancreas, adrenal glands, bilateral kidneys, and spleen. (F and G) Head and neck MRI reported right neck enlarged and clustered lymphadenopathy at levels II, III, IV, and Va and supraclavicular. One of the larger lymph nodes at the right inferior jugular chain showed focal intralesional necrotic foci and heterogeneous enhancement. CT = computed tomography, FDG = 2-deoxy-2-[18F]fluoro-D-glucose, MRI = magnetic resonance imaging, PET = positron emission tomography.

A 58-year-old man, a former smoker, with no history of systemic diseases and food or drug allergies, presented to our ENT outpatient clinic with a 5-month history of recurrent oral ulcers, right neck swelling, and hoarseness. Physical examination revealed multiple ulcers over bilateral buccal areas and tongue and right neck multiple lymphadenopathy (LAP) from levels II to IV with 1.5-cm maximal size. Nasopharyngoscopy indicated multiple ulcerative lesions over the tongue tip, mouth floor, tongue, buccal, hypopharynx (involving the posterior pharyngeal wall and epiglottis pyriform sinus), and larynx (Fig. 1). The pathological report of the biopsy revealed buccal area with an ulcer, right tonsil with poorly differentiated squamous cell carcinoma. Whole-body 2-deoxy-2-[18F]fluoro-D-glucose-positron emission tomography (FDG-PET CT) showed bilateral neck LAP with right neck levels II to V lymph nodes enlarged, measuring up to 2.4 cm, while left neck levels II to III lymph nodes were of normal size (Fig. 2). Focal lesions were not found in the gastrointestinal tract, liver, gallbladder, pancreas, adrenal glands, bilateral kidneys, and spleen. Head and neck magnetic resonance imaging reported a primary tumor at the right tonsil with a maximum diameter of <2 cm, right neck enlarged, and clustered LAP (levels II, III, IV, Va, and supraclavicular), with focal necrotic foci in the right level IV nodes and extranodal extension (Fig. 2).

**Figure 3.**
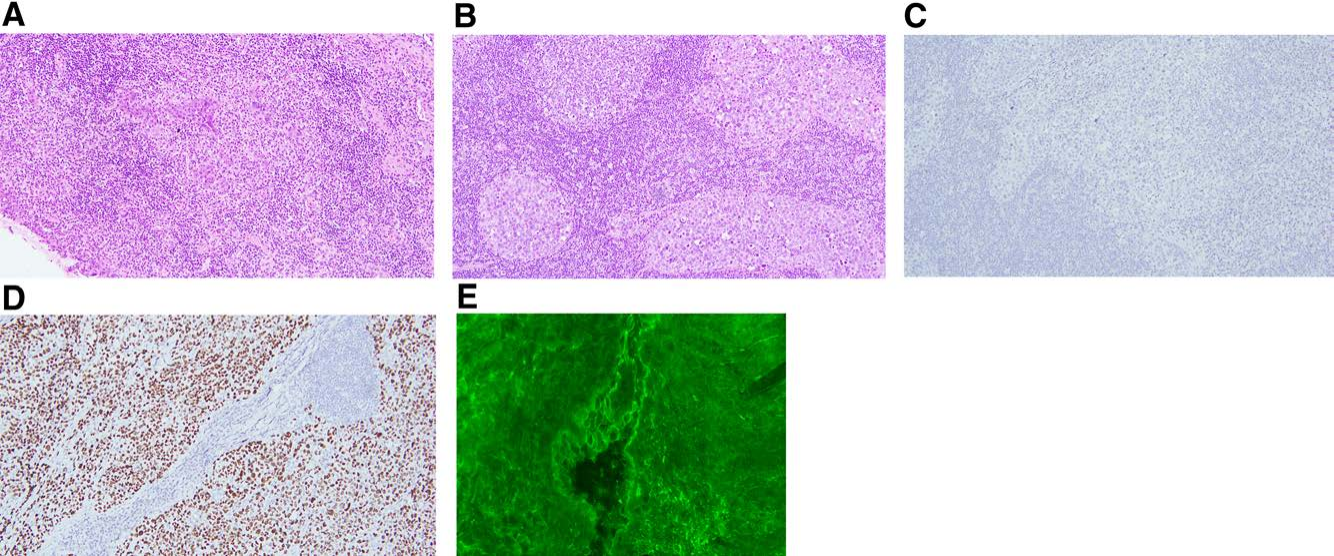
Hematoxylin-eosin-stained and immunohistochemical histopathological images. (magnification: 100×). (A)The tonsil shows nonkeratinizing poorly differentiated squamous cell carcinoma on the superficial portion. The carcinoma is arranged in nests. The carcinoma cells show large vesicular nuclei, distinct nucleoli, and a considerable amount of eosinophilic cytoplasm with moderate to marked nuclear pleomorphism and frequent mitosis. (B) The right enlarged lymph node shows metastatic carcinoma. (C) Negative for p16 in the right enlarged lymph node. (D) Positive for p40 in the right enlarged lymph node. Direct immunofluorescence (DIF; magnification: 400×). (E) Skin DIF shows C3 deposition at the intercellular junctions of keratinocytes.

Simultaneously, we consulted a dermatologist due to the emergence of bullae, ovoid brownish macules, and patches over his trunk. The presence of serological antibodies, including positive anti-intercellular substance antibodies with a titer of 1:40 and negative antibasement membrane antibodies with a titer of < 1:20, suggests a tendency toward pemphigus rather than pemphigoid, particularly in the context of multiple mucosal eruptions and skin bullae. Direct immunofluorescence (DIF) of skin specimens revealed C3 deposition at the intercellular bridges of keratinocytes (Fig. 3). Under the above results, he was diagnosed with PNP induced by the right tonsillar cancer, cT1N3bM0, stage IVB.

**Figure 4.**
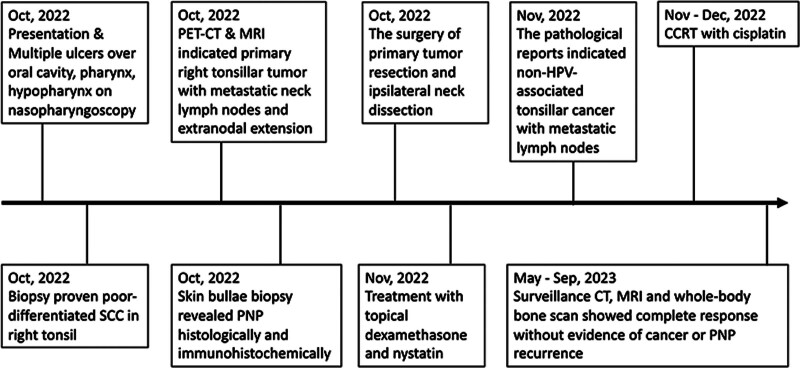
The timeline of his clinical presentation, diagnostic process, treatment, and outcome. CT = computed tomography, CCRT = concurrent chemoradiotherapy, MRI = magnetic resonance imaging, PET = positron emission tomography, PNP = paraneoplastic pemphigus, SCC = Squamous cell carcinoma.

The surgery of primary tumor resection and ipsilateral neck dissection has been performed. The pathological reports indicated that the right tonsil showed nonkeratinizing poorly differentiated squamous cell carcinoma on the superficial portion (Fig. 3). The lymph node showed metastatic carcinoma. Immunohistochemically, the carcinoma is positive for p40 and negative for p16, indicating not HPV-associated (Fig. 3). After surgery, the patient’s skin condition significantly improved, with existing bullae crusting over, and no new vesicles or bullae developed. Topical dexamethasone and nystatin were prescribed to alleviate oral ulcers and prevent oral infections before adjuvant concurrent chemoradiotherapy (CCRT) with cisplatin. The post-CCRT follow-up imaging at 3, 6, and 9 months, which included magnetic resonance imaging, computed tomography, and whole-body bone scans, indicated no signs of cancer or PNP recurrence. A timeline of his clinical course is illustrated in Figure 4.

## 3. Discussion

PNP is a life-threatening autoimmune bullous disease with a mortality rate ranging from 75% to 90% and an approximate 5-year survival rate of about 40%.^[2]^ The pathogenesis of PNP remains unclear at present, but a consistent clinical feature is its inherent association with an underlying malignancy. Malignant cancer is typically detected prior to the onset of PNP although exceptions exist. Among ≈500 reported cases to date, the most common cancers associated with PNP are NHL, followed by Castleman disease and chronic lymphocytic leukemia.^[2]^ To date, only 1 case of PNP related to tonsillar cancer has been reported.^[4]^ In addition, a solitary early case report documented PNP induced by radiotherapy in a patient with NHL.^[5]^

Many autoimmune antibodies causing cellular immune responses have been reported, the most common of whom are against the plakin protein family, including envoplakin, periplakin, desmoplakins I/II, plectin, and bullous pemphigoid antigen.^[6]^ These antibodies target various tissues such as oral mucosa, skin, oropharynx, nasopharynx, esophagus, and respiratory epithelium. In most instances, oral mucosal lesions present as the initial and predominant symptom, followed by the emergence of skin lesions. Oral mucosal lesions can manifest as erosions, ulcers, vesicles, or bullae. Multiple, recurrent, refractory, and painful lesions can extend to the lip, oropharynx, nasopharynx, and hypopharynx. Skin lesions, characterized by considerable variability, may appear as erythema, erythroderma, bullae, erosions, or ulcers. Given the involvement of respiratory epithelium in over 90% of PNP cases, symptoms such as dyspnea, obstructive lung disease, and bronchiolitis obliterans may progress to respiratory failure, ultimately resulting in mortality.^[6]^

PNP often requires differentiation from other autoimmune blistering diseases such as bullous pemphigoid. In this case, the results of serum antibodies, including anti-intercellular substance antibodies positive and antibasement membrane antibodies negative, incline the diagnosis toward pemphigus rather than pemphigoid. Furthermore, the skin DIF examination reveals C3 deposition at the intercellular bridges of keratinocytes, a finding that aligns with the characteristics of PNP.^[7]^ Due to the polymorphic characteristics of PNP, there are still no universally accepted diagnostic criteria. In summary, diagnostic criteria, as proposed by Svoboda et al,^[8]^ Anhalt et al,^[9]^ and Camisa and Helm,^[10]^ encompass clinical presentation, concomitant internal neoplasm, and histological and immunological evidence. The clinical presentation comprises polymorphic mucous lesions with or without cutaneous involvement. Acantholysis, lichenoid interface, and keratinocyte necrosis can be observed in histopathological examination, either in isolation or concurrently. Immunological evidence can be established through the examination of serum antibodies, DIF, indirect immunofluorescence, and immunoprecipitation. Despite the high sensitivity and specificity of positive antiplakin antibodies in immunoprecipitation for PNP diagnosis, the constraints on their availability have led us to employ serum antibodies and DIF as additional immunological diagnostic measures in this case.^[11]^

To date, surgical resection, chemotherapy, and/or radiation therapy to treat occult cancer are the first-line management for PNP. The role of immunomodulation as adjunctive therapy is to prevent acute respiratory mucosal involvement, enhance the patient’s quality of life, and prepare the skin for radiation therapy. Although the majority of patients’ mucosal lesions exhibit resistance to high-dose steroids, high-dose systemic steroids remain the first-line treatment.^[2,12]^ Some studies have indicated that a combination of steroids with immunosuppressants, intravenous immunoglobulin, or plasma exchange can be effective and safe for certain patients. If a patient is refractory to many of the previous treatments, monoclonal antibodies such as rituximab, alemtuzumab, and daclizumab can be considered as potential options.^[2]^ Given that our patient’s mucosal symptoms did not cause respiratory distress, topical steroids and antifungal agents were administered to alleviate oral lesions and prevent secondary infections. In our opinion, effectively managing skin and mucosal symptoms before starting CCRT is of paramount importance. Chemotherapy can lead to immunocompromise in patients, thereby elevating the risk of mucosal or cutaneous microbial infections.

This case, along with the other previously published case of tonsillar cancer-associated PNP, demonstrates a disease-free status during ≈1 year of follow-up. The 5-year survival rate for tonsillar cancer is not significantly different from that of the most common PNP-associated condition, NHL.^[13,14]^ The prognosis of PNP and its potential correlation with the specific type of occult cancer await further validation through the accumulation of additional case data. The treatment effectiveness and subsequent survival of patients should be evaluated individually, considering variables such as cancer type, TNM staging, respiratory complications, and infections.

In conclusion, when physicians encounter multiple, recurrent, refractory, and polymorphic oral lesions, apart from considering head and neck cancer, PNP should also be included in the differential diagnosis. Oral mucous ulcers or skin blisters may offer an opportunity for early detection and address a potential underlying malignancy. While conducting histological examination of oral lesions to determine malignancy, it is also possible to observe histological characteristics indicative of pemphigus concurrently. A comprehensive systematic examination to ascertain the presence of concomitant internal neoplasm is imperative for diagnosing PNP. Serum antibody testing and immunohistochemical antibody staining can further confirm the diagnosis of PNP. Appropriate surgical intervention, postoperative steroid therapy, and infection prevention are feasible and advantageous approaches in the treatment of PNP patients, facilitating subsequent CCRT.

## Acknowledgments

The authors would like to thank Dr Chia-Chin Tsai for assistance with the interpretation of histopathological images.

## Author contributions

**Data curation:** Shih-Chun Lu, Hung-Lun Chu.

**Investigation:** Shih-Chun Lu, Hung-Lun Chu, Hann-Ziong Yueh.

**Writing – original draft:** Shih-Chun Lu, Hung-Lun Chu.

**Writing – review & editing:** Shih-Chun Lu, Hung-Lun Chu, Che-Hsuan Lin.

**Methodology:** Hann-Ziong Yueh.

**Project administration:** Hann-Ziong Yueh.

**Supervision:** Che-Hsuan Lin, Yang Chou.
